# Pericardial Tamponade After Left Atrial Appendage Occlusion Placement: A Case for Emergent Point-of-Care Ultrasound

**DOI:** 10.7759/cureus.79886

**Published:** 2025-03-01

**Authors:** Jonathan Schonert, Joseph Minardi

**Affiliations:** 1 Emergency Medicine, St. Luke's Hospital, Chesterfield, USA; 2 Emergency Medicine, West Virginia University, Morgantown, USA

**Keywords:** cardiac echo, cardiac tamponade, laao, pocus (point of care ultrasound), postoperative pericardial effusion, watchman

## Abstract

The prevalence of atrial fibrillation (AF) is rising, with left atrial appendage occlusion (LAAO) devices emerging as viable alternatives to anticoagulation for stroke prevention in high-risk patients. Although LAAO devices reduce bleeding risks compared to traditional anticoagulants, they carry procedural risks, including pericardial effusion and tamponade.

We present a case of a 70-year-old male with a history of AF who presented to the emergency department with shortness of breath, dizziness, and weight gain five days post-LAAO placement. Point-of-care ultrasound (POCUS) revealed a large pericardial effusion with tamponade physiology. Emergency pericardiocentesis drained 800 mL of fluid, resulting in rapid clinical improvement. The patient’s recovery was uneventful, and he was discharged two days later.

Pericardial effusion and tamponade are serious complications of LAAO devices, often presenting with nonspecific symptoms. Emergency physicians should be familiar with the procedure itself, its complications, and the utility of POCUS in expediting care as seen in this case.

## Introduction

The prevalence of atrial fibrillation (AF) is expected to continue to rise in the population, with some estimates predicting double the incidence by 2035 [1]. With an aging population and increased co-morbidities complicating anticoagulation for stroke prevention, left atrial appendage occlusion (LAAO) devices have emerged as a viable alternative to traditional warfarin or direct oral anticoagulants [2]. For patients at higher risk of bleeding with a CHADS-Vasc score equal to or greater than 2, studies have demonstrated that LAAO is non-inferior to warfarin while offering a lower risk of hemorrhage or death [3]. LAAO devices are gaining popularity, and their use is expected to increase along with the growing prevalence of AF. Emergency physicians must be aware of the procedure and its associated complications, particularly in the immediate postoperative period. Among the most serious complications is pericardial effusion requiring intervention, with reported incidence rates of 1.2-5% [4]. These complications are most common within the first 24 hours but can occur later.

Pericardial effusions and early pericardial tamponade often present with nonspecific symptoms [5]. Classic findings, such as distant heart sounds, dilated neck veins, new cardiomegaly on chest X-ray, or electrical alternans on electrocardiogram are rarely observed in the emergency department [6]. Point-of-care ultrasound (POCUS) can expedite the diagnosis and should be utilized during the initial evaluation of patients with shortness of breath or AF, especially post-LAAO.

This case illustrates the effective use of POCUS in diagnosing and managing a patient with a pericardial effusion following LAAO placement.

## Case presentation

A 70-year-old male presented to the emergency department via ambulance with shortness of breath and a racing heart. He also reported dizziness and a 12-pound weight gain over the past few days.

His medical history was significant for paroxysmal AF, Crohn's disease, and iron deficiency anemia secondary to occult gastrointestinal bleeding. He had been on apixaban and aspirin until five days prior when he underwent LAAO due to an elevated bleeding risk. Post-procedure, his anticoagulation regimen was transitioned to clopidogrel and aspirin. The patient reported being in normal sinus rhythm shortly before the procedure but was unsure when his symptoms began. He denied chest pain.

On examination, the patient appeared mildly distressed. Vital signs were a heart rate of 114 bpm, blood pressure of 97/72 mmHg, normal temperature, and normal oxygen saturation. The cardiac exam revealed an irregularly irregular rhythm. Lung auscultation showed diminished breath sounds without wheezing or rales. Moderate bilateral lower extremity edema was noted.

**Figure 1:**
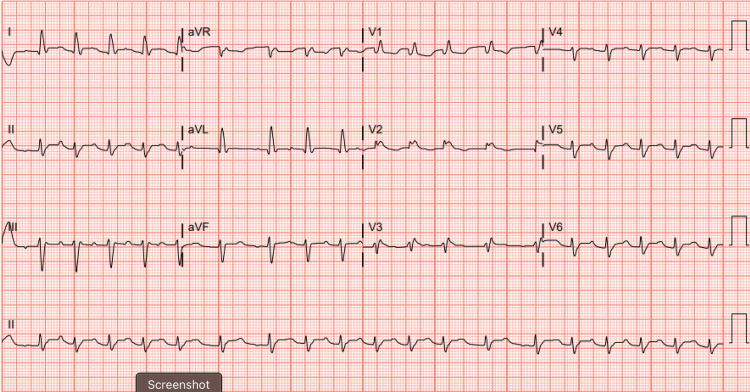
EKG showing pre-existing right bundle-branch block Atrial fibrillation with a rate of 117. Pre-existing right bundle-branch block.

**Figure 2:**
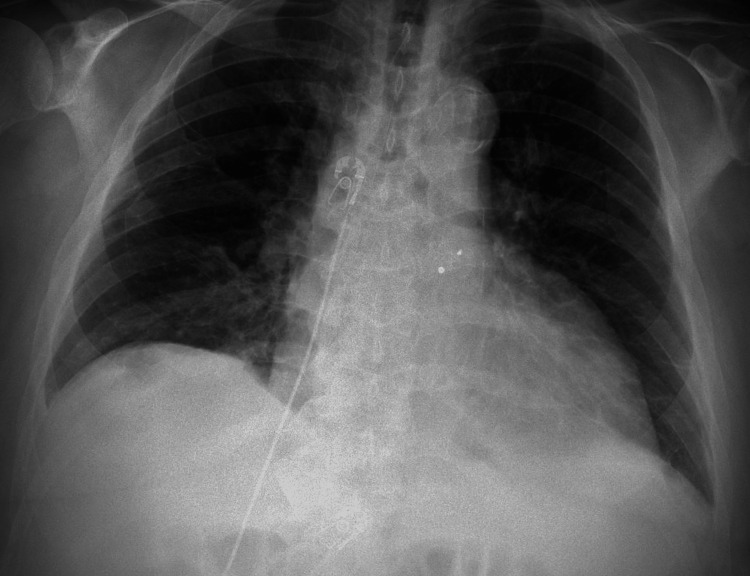
Chest X-ray showing cardiomegaly Official radiologist final impression: No acute process. Cardiomegaly.

The initial EKG (Figure 1) and portable chest X-ray (Figure 2) are shown below. Initial blood work noted a CBC with baseline mild anemia, a creatinine of 1.8, which was new compared to baseline, mild liver enzyme elevations, and a brain natriuretic peptide of 450.

**Figure 3:**
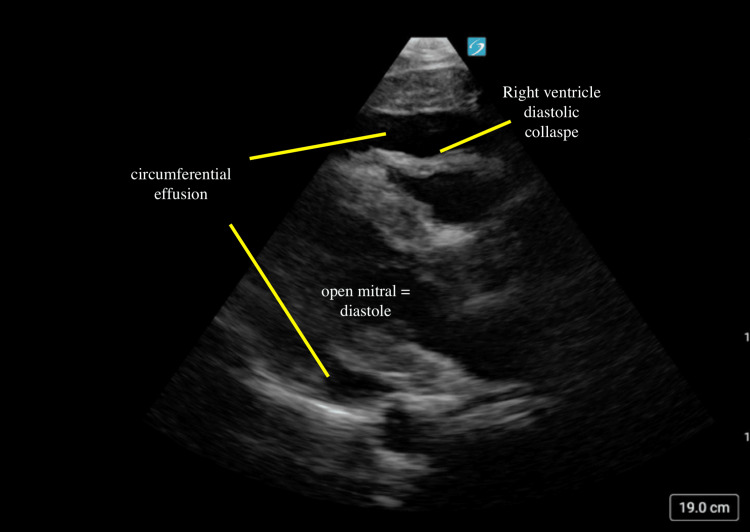
Parasternal long-axis tamponade In this image, a significant circumferential pericardial effusion is seen. Partial collapse of the right ventricle is also seen which occurs during diastole, which is demonstrated by an open mitral valve.

**Figure 4:**
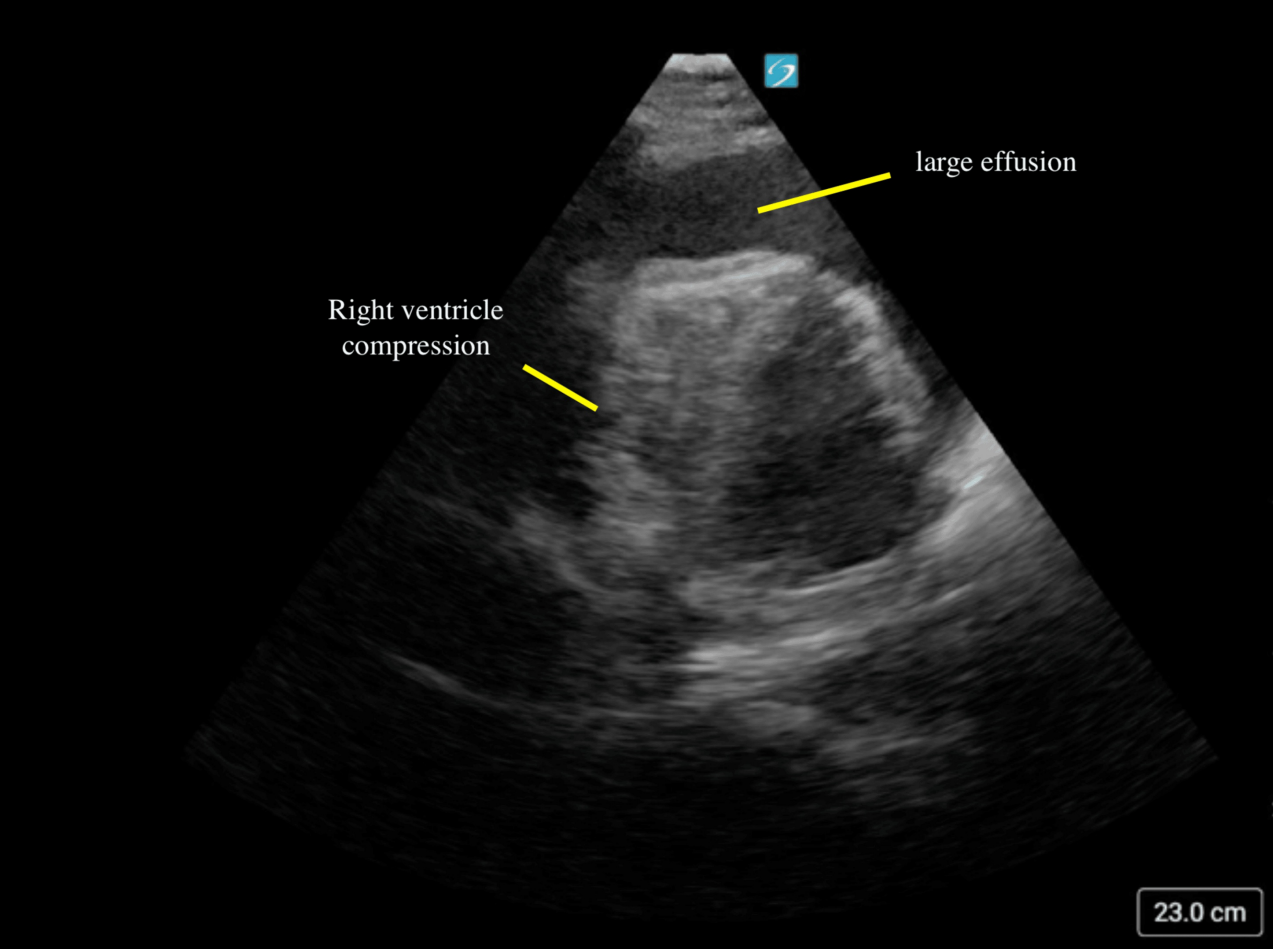
Apical four-chamber tamponade with right ventricular compression In this image, a large circumferential pericardial effusion is again noted. Compression of the right ventricle is also noted.

**Figure 5:**
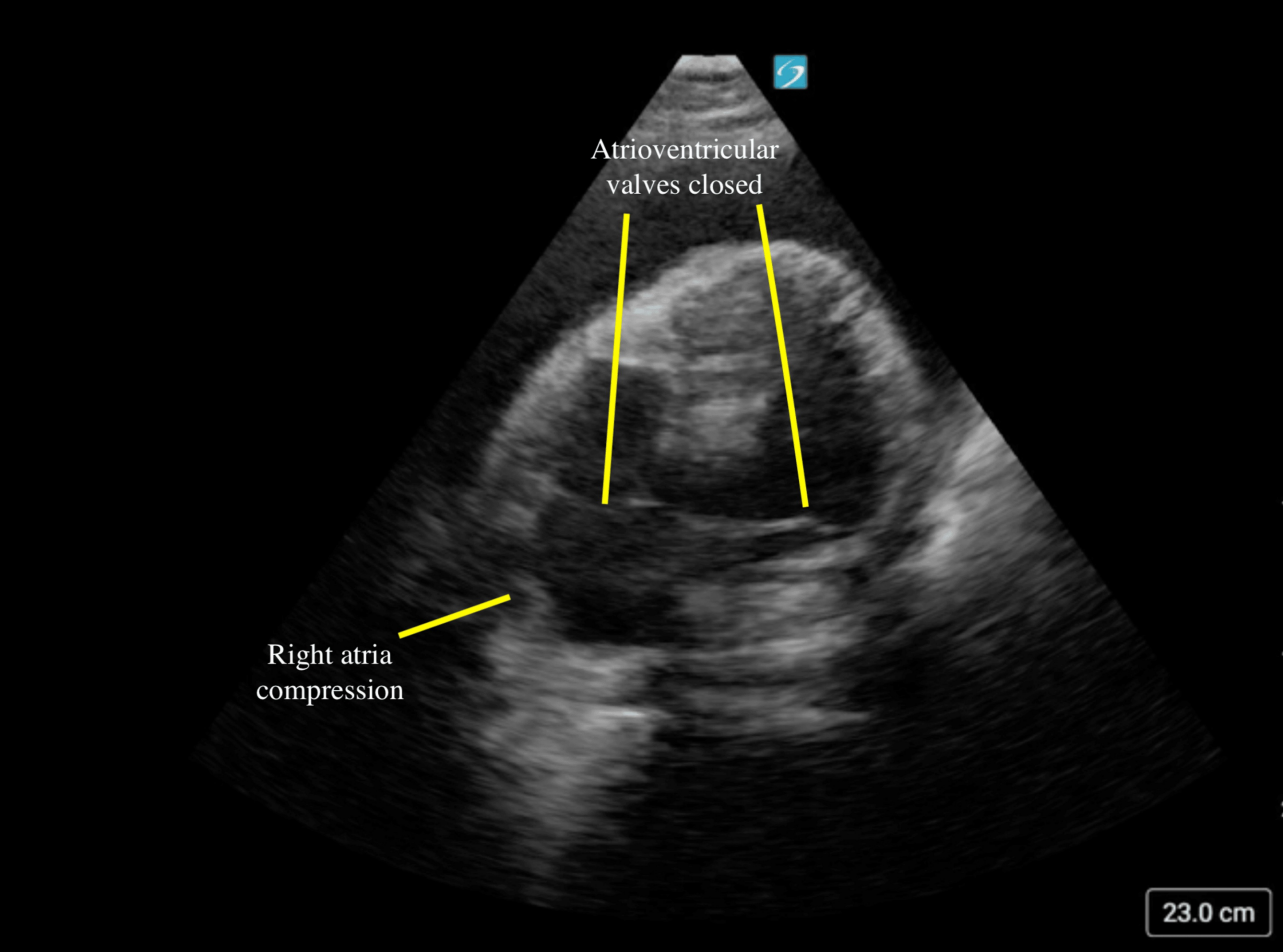
Apical four-chamber tamponade with right atrial compression In this image, a large circumferential pericardial effusion is again visualized. Compression of the right atrium is also noted to occur while the atrioventricular valves are noted to be closed.

POCUS revealed a large pericardial effusion with evidence of tamponade physiology (Figures 3-5; Video 1). Interventional cardiology was consulted, and the patient was taken directly to the catheterization lab for emergent pericardiocentesis. Approximately 800 mL of serosanguinous fluid was drained, and a pericardial drain was placed and removed two days later. The patient recovered well and was discharged home two days later.

**Video 1:** Pericardial tamponade status post left atrial appendage occlusion procedure Multiple clips demonstrating parasternal long and apical four-chamber views of pericardial tamponade physiology with annotation

## Discussion

The left atrial appendage is a common source of thrombi in patients with AF [7]. While surgical management has been an option, it is associated with high complication rates and is typically reserved for patients already undergoing cardiac surgery [8]. Over the past decade, LAAO devices have demonstrated increased success rates with declining complication rates, making them a preferred alternative in eligible patients [9].

The primary indication for LAAO is non-valvular AF in patients with contraindications to long-term anticoagulation. These contraindications include thrombocytopenia, recurrent bleeding, a history of severe bleeding, high fall risk, or the need for dual antiplatelet therapy. Patients typically require anticoagulation or dual antiplatelet therapy for six weeks post-procedure [10].

The LAAO procedure begins with obtaining formal vein access. TEE is then performed to assess for the presence of LAA thrombus before proceeding with device deployment. Once the thrombus is ruled out, a trans-septal puncture is performed to gain access from the right atrium into the left atrium. Under fluoroscopic and TEE guidance, the LAA is measured to determine the appropriate device size, after which the occlusion device is deployed. TEE is used to confirm no pericardial effusion immediately following the procedure and then eventually redone 30-45 days later to confirm device success [11].

Pericardial effusion and tamponade are potential complications due to the thin walls of the left atrium and LAA, occurring most commonly within six hours post-procedure [12]. Diagnosis relies on echocardiography, and tamponade is often a clinical diagnosis [11]. Emergency physicians should maintain a high index of suspicion in patients presenting with any vague symptoms shortly post-LAAO procedure.

POCUS is invaluable in rapidly identifying pericardial effusion and narrowing the differential diagnosis in dyspneic patients. A comprehensive bedside assessment can be done fairly quickly with practice, including both evaluation of cardiac function and lung findings in a limited protocol similar to the bedside lung ultrasound evaluation (BLUE) protocol [13]. The authors are advocates for regularly bringing the POCUS to the patient’s bedside during the initial assessment. Not only can you assess for pericardial effusion, but you can also quickly diagnose or exclude pneumothorax, systolic congestive heart failure, pulmonary edema, large consolidations, and pleural effusions.

Depending on the clinical setting, POCUS is also an invaluable tool to provide real-time guidance for pericardiocentesis. It may be necessary for the emergency physician to perform bedside pericardiocentesis if the patient’s condition demands and/or if consulting services aren’t immediately available. Numerous instructional videos are readily accessible online to provide guidance for physicians navigating this potentially intimidating procedure [14]. Pericardial tamponade following LAAO placement can often be managed conservatively with pericardiocentesis alone; however, in case of persistent hemodynamic instability, thoracotomy may be required [15].

As mentioned above, pericardial tamponade is a clinical diagnosis. The typical findings on bedside ultrasound often reflect either rapid or gradual pressure gradients between the pericardium and the cardiac volume. Key POCUS findings for tamponade include right atrial collapse during ventricular systole, right ventricular diastolic collapse, and lack of respiratory variation in the inferior vena cava [16].

In the author's experience, in respiratory distress patients, adequate views of the heart can still be found even if the patient is sitting up and breathing quickly. The parasternal long and short axes are still fairly straightforward to obtain with patience. In patients who are tachycardic, pausing the ultrasound video and then backtracking the video to view cardiac walls in slower motion or at full end-systole or end-diastole can help to better visualize subtle abnormalities.

## Conclusions

LAAO devices offer promising alternatives to anticoagulation in selected AF patients but come with potential complications that emergency physicians must recognize. Pericardial effusion and tamponade are life-threatening complications that can present with often subtle clinical signs. POCUS should be routinely employed in the initial evaluation of patients with shortness of breath or AF, particularly post-LAAO, to expedite diagnosis and management. This case underscores the critical role of POCUS in diagnosing LAAO-related complications, emphasizing its routine use in post-procedural AF patients.
